# Determining the psychometric properties of safety attitudes questionnaire in NICUs

**DOI:** 10.1186/s40359-023-01229-9

**Published:** 2023-07-20

**Authors:** Arezo Sarhadi, Azam Shirinabadi Farahani, Maryam Rassouli, Malihe Nasiri, Mohadese Babaie, Fatemeh Khademi

**Affiliations:** 1 Department of Pediatric Nursing, School of Nursing and Midwifery, Shahid Beheshti University of Medical Sciences, Tehran, Iran; 2 Department of Biostatistics, School of Nursing and Midwifery, Shahid Beheshti University of Medical Sciences, Tehran, Iran; 3 Department of Nursing, Faculty of Nursing, Arak University of Medical Sciences, Arak, Iran

**Keywords:** Psychometric properties, Safety attitude questionnaire, NICUs, Nurses

## Abstract

**Introduction:**

This study aimed to translate and assess the psychometric properties of the Persian version of the “Safety Attitude Questionnaire” in the NICUs.

**Methods:**

In this psychometric study, the “Safety Attitude Questionnaire” was translated into Persian. Then this version was used for psychometric evaluation. For this purpose, the qualitative face, content validity and construct validity were performed by confirmatory factor analysis. Internal consistency and stability reliability were calculated. Data were analyzed using SPSS and AMOS software.

**Results:**

Face validity was also performed with a slight change in four items. The factor structure of the tool was determined by confirmatory factor analysis. Fit indices were appropriate. Internal consistency reliability in the whole questionnaire was 0.65 and the stability reliability was calculated to be 0.64.

**Discussion and conclusion:**

“Safety Attitude Questionnaire” has appropriate psychometric properties and can be used in NICUs.

## Introduction

The healthcare system has always been a high-risk context in which adverse events are likely to occur [1]. Infants in neonatal intensive care units (NICUs) are the most vulnerable among all hospitalized population groups and experience many adverse events [2, 3]. The prevalence of adverse events among this age group seems to be more severe when infants with lower birth weights and younger fetal ages are hospitalized [4]. Research shows that infants hospitalized in NICUs experience a higher rate of prescribing errors and adverse drug effects compared to infants in other wards and children in other age groups [5]. Therefore, the necessity of providing quality care in neonatal intensive care units is revealed [6]. Because providing quality care ensures neonatal safety, which is fruitful in preventing medical errors, reducing neurological and physical injuries, and thus reducing mortality [7].

Recently, patient safety in all age groups has become a high priority regarding the quality of health care globally [8]. Focusing on safety is also critical to improving the quality of NICUs and ensures better healthcare and patient outcomes [6].

Nurses are in the first line of protecting the neonate’s safety and can directly identify issues that affect the patient’s safety. They are a significant factor in reducing events and their consequences [7]. In other words, nurses play a key role in implementing safety-related factors and improving safe attitudes and safety culture [9].

On the other hand, developing a safety attitude is the basis of achieving safe and quality care. Safety attitude refers to the shared values, perceptions, and behavioral patterns that determine caregivers’ effort, attention, and performance to minimize the insecurity in patients [10]. Therefore, measuring safety attitudes among Nurses is very momentous [11]. If healthcare provider organizations want to enhance patient safety, they must have more information about staff safety attitudes [7, 12].

Moreover, it is difficult and even impossible to change beliefs, attitudes, knowledge, or actions, as the components of *culture*, without receiving some kind of feedback. Therefore, an essential step in creating a safety culture is to develop tools to measure the components of that culture [13].

Safety attitudes can be assessed using psychometrically evaluated questionnaires that measure the collective attitude of the staff in organizations [14]. In other words, organizations need to have specific tools to measure safety culture, with the aim of identifying the weaknesses and planning to provide appropriate interventions [15].

Various tools have been developed to evaluate safety attitudes. The *Safety Attitudes Questionnaire* (SAQ) is one of the most widely used tools [15]. This questionnaire is one of the most frequently used tools for examining patient safety. SAQ has good psychometric properties and its results are associated with clinical outcomes. The analysis of its psychometric properties also indicates appropriate validity and reliability [16]. This tool can also measure safety attitudes while predicting the factors affecting it in clinical settings [17].

Despite the high importance of understanding safety attitude and evaluating it in neonatal intensive care units, where the most vulnerable population is hospitalized, limited studies exist investigating the safety attitude of nurses working in these wards [11, 12]. In addition, there is not any Persian version of the questionnaire appropriate to the type of attitude and culture prevailing in the Iranian healthcare system, which could investigate the safety attitude in the neonatal intensive care unit. Therefore, the aim of this study was to translate and determine the psychometric properties of the Persian version of the “Safety Attitude Questionnaire” in nurses working in neonatal intensive care units.

## Methods

### Study design

This psychometric design [18] was used to evaluate the psychometric properties of the Persian version of the “*Safety Attitude Questionnaire*” among Iranian nursing staff. This study was conducted through a convenience sampling of nurses working in educational Neonatal Intensive Care Units (NICUs) in Tehran, Iran, from March to July 2018.

### Setting

This study was conducted in the most prominent treatment and educational hospitals affiliated with Shahid Beheshti University of Medical Sciences, Tehran, including Mahdiyeh, Mofid, Imam Hossein, Shohadaye Tajrish, and Ayatollah Taleqani. These wards are the largest referral centers for premature neonates. In these NICUs, neonates with life-threatening congenital problems and abnormalities or acute problems related to prematurity are admitted. Of all staff, 210 nurses were working in the NICUs.

### Procedures and participants

In the present study, after obtaining permission from the tool developer via E-mail, the questionnaire was translated into Farsi according to World Health Organization guidelines [19]. In this regards, the original version of the questionnaire was translated into Farsi by a professional translator familiar with nursing concepts and was compared to the English tool by the research team members. The Farsi version was back-translated into English by a professional translator familiar with nursing concepts who, of course, was not aware of the English tool. The back-translated version was submitted to the original developer for the examination of major concepts, words and the meanings of the items. After receiving and applying the developer’s comments, the initial Farsi version was prepared. Then this version was used for performing psychometric evaluation. The tool was tested on the population of nurses working in NICUs for its psychometric properties to be evaluated through measuring face and content validity, construct validity, and reliability including internal consistency and stability.

In This study, the participants selected using convenience sampling. The inclusion criteria were all nurses who were at least in bachelor’s degree in science, had at least one month of working experience in the NICU, and had physical and mental health. Of those who worked in the NICUs of the selected hospitals, 180 nurses met the inclusion criteria and completed the questionnaires. Nurses whose position or place of work was changed by the nursing management during the research were excluded.

### Instruments and measures

The instrument used in this research is the Safety Attitude Questionnaire (SAQ) which consists of 34 items. The items are designed on a 5-point Likert scale in the six dimensions. SAQ has been widely used to examine attitudes toward patient safety. A total score of more than 75 is considered a positive attitude [20]. SAQ dimensions definitions [21] are as follows:

- ***Teamwork Climate*** means perceived quality of collaboration between personnel (*e.g., I have the support I need from other personnel to care for patients*).

- ***Job Satisfaction*** refers to positive perception about the work experience and motivation to perform tasks (*e.g., I am proud to work in this clinical area*).

- ***Safety Climate*** deals with perceptions of a strong and proactive organizational commitment to safety (*e.g., I am encouraged by my colleagues to report any patient safety concerns I may have*).

- ***Perceptions of Management*** describe the approval of managerial action (*e.g., Management supports my daily efforts*).

- ***Stress Recognition*** is the acknowledgment of how performance is influenced by stressors (*e.g., I am more likely to make errors in tense or hostile situations*).

- ***Working Conditions*** include aspects of the perceived quality of the work environment and staffing/equipment support (*e.g., The levels of staffing are sufficient to handle the number of patients*).

The second part of the instrument was the demographic questionnaire consisted of information on gender, marital status, working experience, employment status, work shift, level of education, organizational position, courses taken on infant safety and having a second job.

#### Content validity

For measuring the qualitative content validity, the SAQ was provided for ten experts in the field of tool development and nursing to assess the quality of the tool and the appropriateness of the items for nurses working in NICUs. These experts were selected using purposeful sampling and were not part of the research team.

#### Face validity

In order to examine the qualitative face validity, the researcher conducted in-person interviews with 15 selected nurses working in NICUs and asked them to complete the tool and discuss any needs to make changes in the phrasing of the items and share their ideas about the understandability of the questionnaire and its ease of completion.

#### Construct validity

The confirmatory factor analysis (CFA) performed to assess the construct validity. This technique determines the goodness-of-fit between a hypothetical model and data obtained from research samples [22]. The maximum likelihood estimation (MLE) was used to estimate the parameters. Several disparate indices should be used to decide the model’s suitability [23]. In this study, Chi-square, Root Mean Square Error of Approximation (RMSEA), Non-Normed Fit Index (NNFI), Comparative Fit Index (CFI), and Goodness of Fit Index (GFI) used to confirm the dimensions. Since no consensus exists between researchers in the CFA sample size, at least ten subjects recommended per factor [24, 25]. Also, the researchers are advised using 100 to 200 samples while evaluating a tool with more than three factors [26, 27].

After obtaining the necessary permissions, by visiting the desired wards on various days and during different shifts, the researcher explained the objectives of the research to the research subjects, distributed the SAQ and the demographic questionnaire among them, and collected the completed questionnaires. Sampling took three months, from March to July 2018. The subjects were provided with the researcher’s contact number in case they came across any questions in regard with the questionnaires.

#### Internal consistency and stability reliability

The internal consistency reliability assessed using the construct validity data. Cronbach’s alpha was calculated for the total instrument and its dimensions. Cronbach’s alpha value greater than 0.8 is considered excellent, between 0.6 and 0.8 is good, and less than 0.6 is poor [28]. The values above 0.6 are also accepted [29].

The test-retest was conducted to evaluate the stability. Of the participants, 25 nurses [30] were selected using purposeful sampling and asked to complete the questionnaire twice with an interval of 14 days. Then the Interclass Correlation Coefficient (ICC) was calculated. ICC values less than 0.5 indicate poor, between 0.5 and 0.75 indicate moderate, and between 0.75 and 0.9 indicate good reliability [31].

### Data analysis

The descriptive and analytical statistical analyses were performed using SPSS V25. The Kolmogorov-Smirnov was used to test the normality of data. Confirmatory factor analysis was done by AMOS V21. All the tests were done at a significance level of 95%.

## Results

The descriptive analysis results of the data obtained from 180 nurses participating in the study indicated that most respondents were women (96.11%), Married (56.67%), and 88.33% had bachelor’s degrees. Of the participants, 18.89% had a second job and 86.11% received any educational courses on infant safety. Other demographic characteristics of the nurse are shown in Table 1.

**Table 1:** Demographic characteristics of nurses working in NICUs (n = 180)

Variable		Frequency	percent
Working experience	Less than 6 months 6 to 11 months 1 to 2 years 3 to 4 years 5 to 10 years 11 to 20 years 21 years or more	3 93 30 13 29 11 1	1.67 51.67 16.66 7.22 16.11 6.11 0/56
Work shift	Fixed Rotating	56 124	31.11 68.89
The level of education	Bachelor’s degree Master’s degree	159 21	88.33 11.67
Organizational position	Nurse Head nurse	166 14	92.22 7.78

After the translation and back-translation, the three items of “*I would feel safe being treated here as a patient.*”, “*Medical errors are handled appropriately in this clinical area.*” and “*I receive appropriate feedback about my performance.*” underwent slight changes, which became “*I feel safe here if I am treated as a patient.*”, “*Here medical errors are correctly identified*.” and “*I get the right feedback on my performance.*”, respectively.

The opinions of experts in the field of tool development and nursing were used to measure the qualitative content validity. According to this survey, changes were made in the phrasing of four items of “*Medical errors are handled appropriately in this clinical area.*”, “*The culture in this clinical area makes it easy to learn from the errors of others.*”, “*I experience good collaboration with pharmacists in this clinical area*” and “*Communication breakdowns that lead to delays in delivery of care are common*”. The new items are “*Here, medical errors are identified correctly.*”, “*Friendly cooperation in this ward facilitates learning from errors.*”, “*We have good collaboration with clinical pharmacists here.*” and “*The lack of appropriate relationship between the staff is one of the most important factors of disorganizing patient care.*”, respectively. While measuring the qualitative face validity, no changes were made due to the clarity of all the items according to the participants’ opinions.

**Fig. 1:**
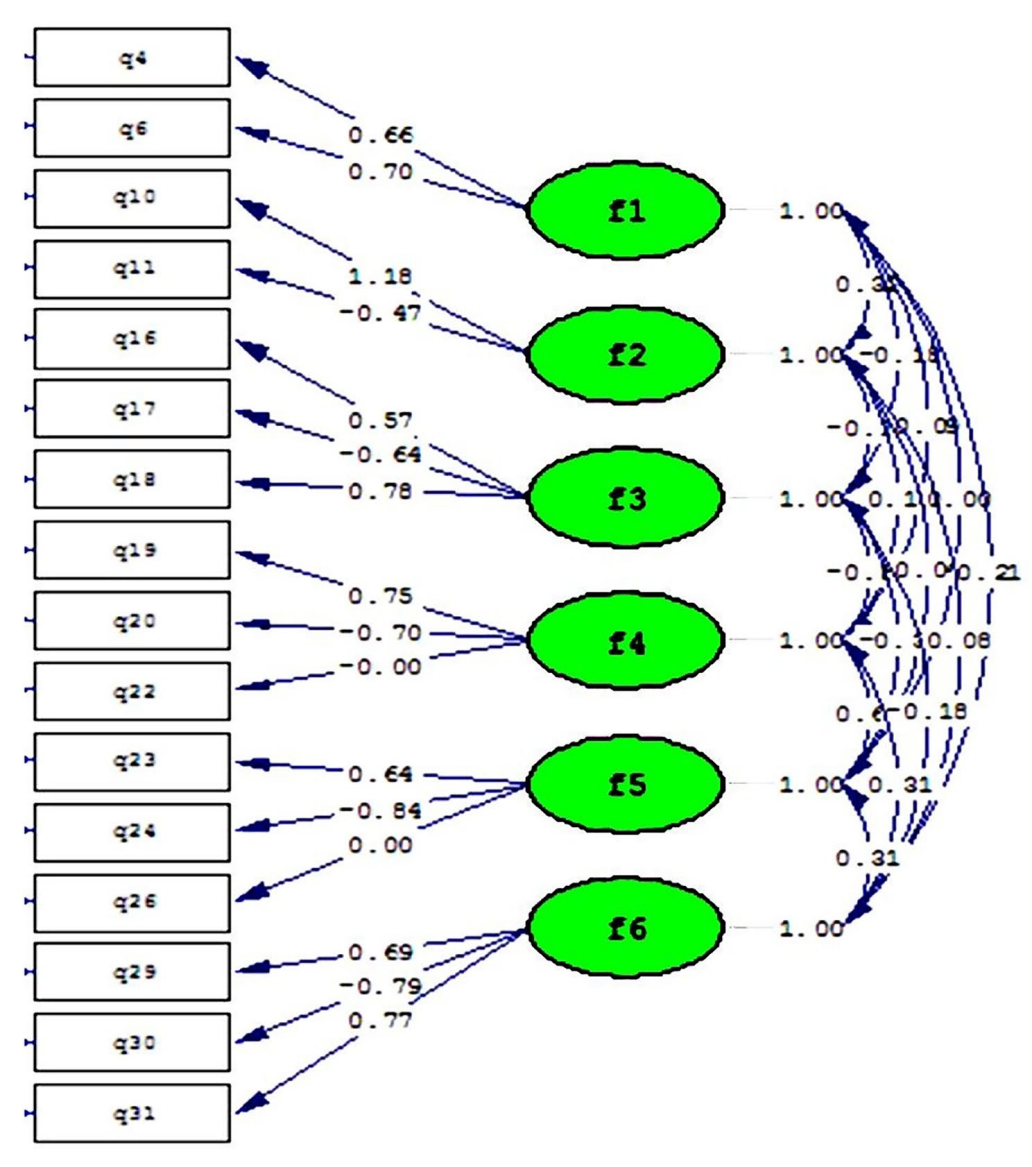
The structure of items model in the Farsi version of SAQ

Confirmatory factor analysis was used to determine the factor structure of the tool. The structure of the Farsi version of SAQ is shown in Fig. 1, in which the dimensions 1 to 6 show *Team Work Climate*, *Safety Climate*, *Job Satisfaction*, *Stress Recognition*, *Perception of Management* and *Working Condition*, respectively. The CFA results for the 6-factor model based on Fig. 1 demonstrate that all dimensions have an acceptable correlation coefficient with related items. Also, based on the T-value test under AMOS software, all the relationships between the dimensions and their items are significant, and there is no heterogeneity. In general, it can be said that the desired model and its constituent concepts are acceptable according to the fit indices of χ^2^/df < 2 [32], RMSEA ˂ 0.08, CFI ≥ 0.90, GFI ≥ 0.90 [33], and NNFI ≥ 0.90 [34] (Table 2), and it is confirmed by six factors in nurses working in neonatal intensive care units.

**Table 2:** The fit indices of the Farsi version of SAQ based on the CFA

Fit index	The obtained value	The acceptable value
Chi-square/df	1.41	< 2
Root Mean Square Error of Approximation (RMSEA)	0.052	˂ 0.08
Non-Normed Fit Index (NNFI)	0.93	≥ 0.90
Comparative Fit Index (CFI)	0.95	≥ 0.90
Goodness of Fit Index (GFI)	0.91	≥ 0.90

The values of Cronbach’s alpha coefficient in all the dimensions of the Farsi version of SAQ were calculated to determine the internal consistency reliability and ranged between 0.67 and 0.83 for the six dimensions (Table 3). Moreover, to measure the stability reliability, the Interclass Correlation Coefficient was calculated at 0.64.

**Table 3:** The internal consistency reliability (Cronbach’s α) for the whole scale of SAQ and its dimensions

Dimensions	Cronbach’s α
Team Work Climate	0.67
Safety Climate	0.72
Job Satisfaction	0.83
Stress Recognition	0.70
Perception of Management	0.75
Working Condition	0.71
Total	0.65

## Discussion

The aim of this study was to translate and determine the psychometric properties of Safety Attitudes Questionnaire in the NICUs of selected hospitals affiliated to Shahid Beheshti University of Medical Sciences with the participation of 180 NICU nurses.

All the individuals participating in the process of translation and back-translation were familiar with the concepts of the tool, and the source and destination languages. After providing the initial Farsi version of the tool, the next steps of the psychometric evaluation were examined. In this study, the WHO procedure was implemented for translation and back-translation. The same procedure was used by Gambashidze et al. (2020) for the translation of SAQ into Georgian, by Carvalho et al. (2012) for the translation and the evaluation of its psychometric properties in Brazil, by Deilkås et al. (2008) its translation into Norwegian, and by Kaya et al. (2010) in the Turkish version of the questionnaire. Among all translation methods, using the translation and back-translation approach recommended by the WHO indicates the significant effort made to ensure the accuracy of the translation [35].

In the Farsi version of SAQ, after translation and back-translation, some changes were made in the 3 items *7*, *8*, and *10*. This is considered normal while translating a tool into the official language of another country. In the case of item 7, the change was related to the temporal tense of the sentence and no words or concepts were changed. In regard with item 8, the original item focuses on *appropriately handled medical errors*, while in the back-translated version it was proposed as c*orrectly identified medical errors*. And in the item 10, the word *appropriate* was replaced with the word *right*. In the study by Devriendt et al. (2012), item 12 was rephrased while using this questionnaire in Belgium, aiming to create cultural compatibility and adaptability with Belgian hospitals [36].

In the study by Gabrani et al. (2015), in order to use the SAQ in Albanian hospitals, it was first translated and back-translated. Then the content validity was approved as acceptable by experts in terms of its relevance, appropriateness and significance in Albanian culture [37].

In order to examine content validity in the present study, the questionnaire was provided to 10 faculty members with expertise in the field of tool development and nursing who were selected through purposive sampling. The experts made a qualitative examination of the relevance or the representativeness, the clarity and the comprehensiveness of the items. Finally, their opinions were applied and changes were made. To evaluate the face validity, the questionnaire was distributed among 15 nurses of the target population through convenience sampling. The clarity and simplicity of phrases and nurse’s understanding and recognition of the items were examined. According to them, the items were understandable and no changes were required.

The same procedure was done by Carvalho et al. (2012) in their study with the aim of translation and the cultural adaptation of this questionnaire in Brazil. After the translation and back-translation of the questionnaire by a group of experts, the questionnaires were distributed among 10 experts in the selected hospitals in order to evaluate the face validity and discover whether the questionnaire is understandable and to make an estimation of the time needed to complete it. After the evaluation, all the items were approved and none were changed [38].

In the study by Devriendt et al. (2012), no major changes were suggested by the two nurses and the two physicians who examined the face validity of SAQ. Minor recommendations have been made for improving the clarity of the phrases. For instance; the word *medical error* has been clarified by adding examples such as falls, medication errors and needle stick injuries. In addition, several linguistic errors were identified and corrected [36].

To examine the construct validity, the Farsi version of the questionnaire was provided to 180 NICU nurses and the obtained data was used in confirmatory factor analysis. The fit indices reported by the confirmatory factor analysis fell in an acceptable range and the fit of the model was confirmed. Therefore, there was no need to remove or change the dimensions or the items of the research tool in the studied population and the construct validity of the Farsi tool was confirmed with the same 6 dimensions as the original questionnaire consisting of *teamwork climate*, *safety climate*, *job satisfaction*, and stress *recognition*, *perception of management* and *working conditions*.

In the study by Nguyen et al. (2015) the structure of the Italian version of SAQ was also examined using confirmatory factor analysis. The questionnaire as a whole had a significant Chi-square index (P < 0.001). The other indices were also acceptable, falling within a range appropriate for the model to fit the data. In this study as well, all the dimensions of the original questionnaire were confirmed [39].

In the study by Göras et al. (2013), in which the Swedish translation of the questionnaire for operating rooms was psychometrically evaluated, confirmatory factor analysis was used to examine the construct validity. The SRMR value was reported to be 0.055, the RMSEA below the recommended value of 0.05 and the CFI much higher than 0.95, which indicates the good model fit approximation of the Swedish version of SAQ [40].

The above studies have confirmed the model and the fit indices. However, in the study by Gambashidze et al. (2020) the six-factor model of the Georgian version of this questionnaire was not confirmed by the confirmatory factor analysis which was performed to determine the construct validity (χ2 / df = 2.14, RMSEA = 0.06, GFI = 0.83, CFI = 0.88, TLI = 0.86), so an exploratory factor analysis was followed. The result of this factor analysis resulted in a modified four-factor model with an acceptable model fit (χ2 / df = 2.09, RMSEA = 0.06, GFI = 0.88, CFI = 0.93, TLI = 0.91). The two dimensions *teamwork climate* and *safety climate* were merged to form the combined dimension named *teamwork and safety climate*. The three major dimensions *job satisfaction*, *stress recognition* and *perception of hospital management* remained in the model while retaining all or most of their key items [41].

- The dimension *teamwork climate* covers the items 1 to 6 [42] and is related to understanding the quality of cooperation between the staff [40]. In a study, poor teamwork accounted for 9% of errors reported in the NICU environment [43]. In maternity wards, poor teamwork was the leading cause of death and injuries caused by delivery in 55% of cases. Team performance is important especially during emergencies where the rescue team must assemble quickly, communicate clearly, and cooperate effectively. Thus, the teamwork climate is an important factor in examining safety attitudes [15].
- The dimension *Safety Climate* consists of items 7 to 13 [42], which focus on perceiving a strong and active organizational commitment to safety [40]. The safety climate assesses perceptions related to an active and strong organizational commitment to patient safety [44, 45].
- *Job Satisfaction* dimension covers items 15 to 19 [42] and is about a positive working [40]. In the study by Gambashidze et al. (2020), job satisfaction was considered one of the most important dimensions that remained in the model by retaining all the main items and was effective in the SAQ.
- The “stress recognition” dimension is associated with items 20 to 23 [42] which are about confirming how stress impacts functioning [40].While translating this questionnaire into Chinese by Lee et al. (2010), this dimension was removed from the final Chinese version of the questionnaire because its relationship with the safety culture was significantly weaker than the other five dimensions were [14]. In addition, in the study by Göras et al. (2013), the *Stress Recognition* dimension had no significant relationship with the dimensions *Teamwork Climate*, *Perception of Management* or *Job Satisfaction* [40].
- The dimension *perception of management* includes items 24 to 29 [42] and pivots around confirming managerial actions [40]. In the study by Deilkås et al. (2008) this dimension was divided into two separate dimensions, one regarding hospital management (top) and the other about local management (unit), the two dimensions were named as the *perceptions of hospital management* and the *perceptions of unit management* [46]. In the study by Profit et al. (2012), the results showed that advances in the recognition and perception of management may be the first step in taking a series of actions in an NICU to improve clinical outcomes and patient safety [15].
- The *working condition* dimension covers items 30 to 32 [42] and refers to the perceptual quality of the working environment and logistic support (staff, equipment, etc.) [40]. A five-year monitoring program by Baldo et al. (2002) discovered that nurses are responsible for 78% of medical errors. Studies show that negative factors in the workplace such as job contradictions, long working hours and care responsibilities’ difficulties play an important role in regard with this issue [47].

In this study, in order to determine the internal consistency, the Cronbach’s alpha was calculated for each dimension and for the whole tool using SPSS V25. The alpha coefficient for all the dimensions fell in the good range. Although the “*Teamwork Climate*” dimension had a lower Cronbach’s alpha than the others, it was still acceptable. This result was similar to a Chinese study. Cronbach’s alpha and Pearson correlation coefficient were measured to examine the internal consistency of the Chinese version of the SAQ in the study by Li et al. (2017). Cronbach’s alpha was reported to be 0.91 for the whole tool and ranged between 0.66 and 0.91 for the 6 dimensions [28]. In the main psychometric study of this instrument [16], although the total alpha was 0.87, “Teamwork Climate” had a lower Cronbach’s alpha (0.62) compared with the other dimensions, which is similar to the present study. In fact, in the original version of this tool, two dimensions of “Teamwork climate” and “Working conditions” had alpha values of 0.62 and 0.63, respectively.

In addition, in the study by Gambashidze et al. (2020) the dimensions of the questionnaire had good internal consistency with a Cronbach’s alpha greater than or equal to 0.7, except the dimension “*working condition*” [41]. On the other hand, there are also studies that, even though the total Cronbach’s alpha was good, some dimensions were not accepted. In this regard, it could refer to the study of Nilsson et al. (2018) and Smits et al. (2017), in which Cronbach’s alpha values were 0.51 to 0.76 and 0.49 to 0.86, respectively [1, 48]. In the study by Nguyen et al. (2015), Cronbach’s alpha for the whole tool was 0.85, implying a good internal coherence. Cronbach’s alpha coefficients for each factor fell between 0.70 and 0.86 [49].

In this study, the test-retest used to determine the stability reliability, and ICC calculated which is accepted by the COSMIN checklist [50]. This value was acceptable, too.

In the study by Nguyen et al. (2015) and in the Italian version of this questionnaire which confirms the seven-factor model, the retest test approach was used to examine the reliability. Pearson correlation showed high consistency between the items on the two occasions of performing the test [39].

In the study by Deilkås et al. (2008), for the Norwegian version of the SAQ, the stability reliability was examined in hospital’s radiology lab, and the ICC was reported to be greater than 0.7 for five of the seven factors, excluding the dimensions *stress recognition* and *perception of management* [46].

## Conclusion

According to the results obtained by the translation procedure, the evaluation of the psychometric properties and the statistical analysis of the Farsi version of Safety Attitudes Questionnaire (SAQ), this version of the questionnaire can be used in NICUs with the aim of assessing the safety attitudes among nurses in these units. Therefore, it is recommended to conduct researches in the future to investigate the psychometric properties of SAQ amongst other health care staff, with larger samples and to study other wards of the hospital as well.

## Data Availability

Due to privacy concerns, the transcripts of the interviews are not available to the public. On reasonable request, the corresponding author can provide transcript information.

## Abbreviations

CFA: Confirmatory Factor Analysis
CFI: Comparative Fit Index
GFI: Goodness of Fit Index
ICC: Interclass Correlation Coefficient
MLE: Maximum Likelihood Estimation
NICUs: Neonatal Intensive Care Units
NNFI: Non-Normed Fit Index
RMSEA: Root Mean Square Error of Approximation
SAQ: Safety Attitude Questionnaire
